# How is COVID-19 Being Contained in China? An Observational Study on the Local Level

**DOI:** 10.1017/dmp.2020.491

**Published:** 2020-12-22

**Authors:** Ziqiang Han, Xin Ai, Justin Veuthey

**Affiliations:** 1 School of Political Science and Public Administration, Shandong University, China; 2 Center for Crisis Management Research, Tsinghua University, China; 3 School of Social Science, The University of Manchester, United Kingdom; 4 Department of Sociology, University of Geneva, Switzerland

**Keywords:** COVID-19, China, local government, emergency response, physical distancing

## Abstract

****Objective**:**

This article investigates how a Chinese local government responded to the ongoing coronavirus disease 2019 (COVID-19) outbreak, and the lessons can be valuable for the ongoing response efforts worldwide.

****Methods**:**

This case study used primarily participant observations and interviews with stakeholders.

****Results**:**

We find that nonpharmaceutical interventions, especially the physical distancing measures, are the primary tools used to contain the spread of the virus, and these actions keep changing to adapt to the situation of the local cases and the directions from the central government. Medical countermeasures, maintenance of essential public services, and effective public communications tactics are important allies of the strict physical distancing measures, which can enforce the public’s compliance.

****Conclusions**:**

Local governments are the frontlines of emergency response. Both regulated policies (eg, physical distancing) and supportive services (eg, medical and essential living support) are necessary to the success of public health emergency response.

The coronavirus disease 2019 (COVID-19) outbreak that began in Wuhan, China, has quickly become an ongoing global pandemic.^1^ China suffered a massive outbreak with 84,373 confirmed cases and 4643 deaths by April 30, but fortunately, the spreading was slowed down and contained by early March.

As the first country with a massive outbreak, China has taken robust physical distancing measures to flatten the curve. Since the first 27 cases of coronavirus pneumonia were officially reported on December 31, 2019, Chinese authorities invested heavily in scientific research and epidemiology investigation of the novel virus. Once the human-to-human transmission of the coronavirus was confirmed on January 20, the national government swiftly implemented rigorous social control measures. The city first hit by the outbreak, Wuhan, with a population of more than 10 million, was entirely locked down on January 23, just 3 d after the confirmation of human-to-human transmission. Shortly after that, the lockdown was expanded to the whole Hubei Province and then applied across the entire country. With such strong physical distancing and strict contact tracing measures, the disease’s spread was quickly slowed down by February. Since early March, the daily new confirmed cases had been reduced to around 100, and most of them are either imported cases or asymptomatic patients, with several occasionally small outbreaks in cities, such as Beijing, Qingdao, etc.

Lessons can be learned from China’s response to the COVID-19. Indeed, the epidemiology (eg, how it is transmitted) and clinical features, as well as the screening, symptoms treatment, and control measures, have been changing with new insights of this pandemic.^2^ For example, in many Western countries, scientists and authorities, such as the United States Centers for Disease Control and Prevention (US CDC) advised against the general public wearing face masks as a protective action. Nevertheless, they eventually changed their viewpoint, and the US CDC started to suggest using face masks as a precautious protection measure on April 4 as the disease continued to be escalated.^3^

There is no in-depth study about how the COVID-19 was being contained in China, especially from the public administration perspective, in addition to some superficial discussions^4^ or news reports.^5^ Most of the current publications regarding COVID-19 lessons are modeling-based epidemiology,^6^ and there is an urgent need for first-hand observations and reports from the field. Thus, this article contributes such knowledge by describing and analyzing a local government’s response to the COVID-19 using our participatory observations and rich firsthand data. This article studies the response of “T County,” a county sitting on the border with Hubei Province, to contribute the knowledge of public health emergency management and the containment of the ongoing COVID-19 pandemic.

Local governments at the county level are internationally the frontlines of emergency management,^7^ and this is also the case in China.^8-10^ During emergencies, local officials are usually responsible for emergency response, and they need good communication skills, “big-picture” thinking, and the capacity to adapt to administration and politics^7^ and even the ability for “improvisation.”^11^

This article focuses on the emergency response and early recovery period while being mindful that the COVID-19 pandemic is ongoing. Emergency management can include different stages from the time dimension. The traditional way is to divide the whole cycle into 4 phases, including the mitigation, preparedness, response, and recovery,^12^ while the crisis approach separates it more detailed into early detection, sense-making, critical decision-making, coordination, meaning-making, accounting, and performance and lessons learning processes.^13^ The response and recovery stages have been and will continue to be important but challenging issues of emergency management because our modern society is becoming more interdependent and complex,^14^ and there is an increasing expectation of better leadership in the public sector from the public.^15,16^ Thus, this article proposed a 3-stage framework from the theories mentioned above for analysis, and the 3 stages are the sense-making and warning, coordinative response, and early recovery with cautiousness.

Public health emergency management has unique features compared with the emergency management of natural-induced and human-made disasters.^17^ One example is the phenomenon called role conflict and “role abandonment.” This phenomenon is not common during natural-induced disasters, but it has a higher probability of occurring in public health emergencies, and this is particularly the case during infectious disease outbreaks.^18^ Other significant differences are the components and capacities needed for response. The capabilities of public health emergency response proposed^19^ are different from the Emergency Support Functions (ESF) defined in the National Response Framework,^20^ although there are overlaps. In addition to the essential emergency response functions such as coordination, medical and health-related capacities like laboratory testing, public health surveillance and epidemiological investigation, and nonpharmaceutical intervention are particularly necessary for public health emergency response.^19^

This research analyzes a local COVID-19 response and recovery case in China using a model including 3 stages and 5 components (Table 1). The 3 stages are the (1) sense-making and warning, (2) the coordinative response, and (3) the early recovery with cautiousness. The 5 components include (1) the emergency declaration and the establishment of the emergency response team, (2) the nonpharmaceutical interventions, (3) the medical and health measures, (4) essential public service maintenance, and (5) public communications.

**Table 1.**
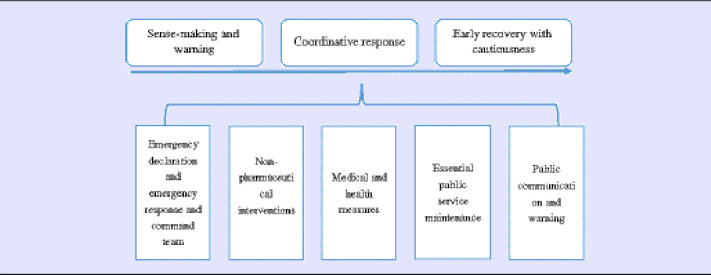
The analysis framework

## Methods

### Background of the Selected County

**Figure 1.**
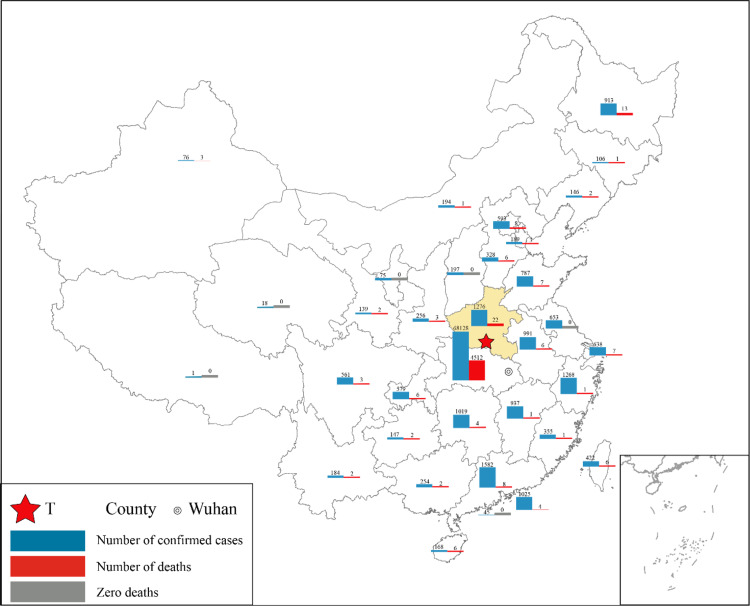
The study place and the prevalence of COVID-19 in China (data updated on April 20).

T County is located in the Southwest of Henan Province and bordered with Hubei Province (Figure 1). The capital of Hubei Province, Wuhan, was the epicenter of the COVID-19 outbreak in China and is the nearest big city to T County. The distance from Wuhan to T County is 278 km. Many citizens of T County are either working or studying in Wuhan, and there are railways and highways connecting the 2 places.

There are 16 towns in T County, with a total population of 470,000. T County was identified as 1 of the poorest counties by China’s central government in 1985 and is still 1 of the poorest today. T County is a typical inland county of contemporary China with a mix of traditional culture and modern lifestyles. Although many young people have left to find work in big cities, most of them choose to return to T County during the Spring Festival holiday each year. Generally, the official Spring Festival holiday starts 1 d before the Spring Festival (January 25, 2020) and lasts for 7 d, and the Spring Festival Eve is the time for all family members to gather together (January 24, 2020) like Christmas Eve in Western cultures. For residents living in the county, the Spring Festival always means an almost 1-mo-long break. For most urbanites, it usually lasts at least 2 wk. Most families prepare lots of food for themselves and visitors, and the most common activities during the Spring Festival holiday are visiting and having dinner with extended family members, friends, neighbors, and sometimes even work colleagues.

Regarding the health services capacities, there are 3 large hospitals within the county. Two of them are privatized, and 1 is public. The public hospital was designated as the official treatment hospital for the severe acute respiratory syndrome coronavirus 2 (SARS-COV-2) patients and other patients with fever symptoms during this outbreak. There are 386 employees and 600 beds in the public hospital. Since this hospital was designated as the official county center for fever symptoms, the hospital had accepted over 2000 patients by March 11, and treated 40 SARS-COV-2 like patients, including 21 confirmed positive cases.

The county had already experienced another significant public health emergency in 2017 after the 2003 SARS epidemic. One confirmed H7N9 infection was identified in a poultry market in T County, and the local government, thus, had the experience of responding and working with the Chinese CDC experts from the provincial government.

### Data Collection and Analysis Methods

Participatory observations and informal interviews are the primary research method adopted in this research. Some of the authors experienced the whole emergency response period as general residents. That said, some of the authors had participated in the local government’s coordination meetings, and some of us had volunteered for the community response teams. Notes and photos were taken to record the critical scenarios and situations. Simultaneously, informal interviews with 2 emergency response team members of the county government, 2 doctors of the hospital, 3 street-level officials of township government, 5 volunteers of community emergency response teams had been conducted. Moreover, we collected all the local policies and news reports from the county government’s website, social media account, and a local news portal. In total, all of the 12 orders and 95 public communication briefs (PCB) from the county government, and 21 news reports about the local leaders’ activities were collected for analysis.

## Results

The COVID-19 virus infected 21 individuals within T County and all of them recovered. The first 3 cases were confirmed at the same time on January 28, which is 8 d after the confirmation of human-to-human transmission in Wuhan, while all the infected cases had been identified on February 10. The first recovered case was on February 4, and the last patient recovered on March 1, and there were no new cases after that. There were 1276 confirmed COVID-19 cases within the Henan Province, 22 of them perished, and the rest recovered. The first case within Henan Province was confirmed on January 21, 1 d after the confirmation of human-to-human transmission, and the first death was on January 25, while the first recovery was on January 28.

The authors differentiate the COVID-19 response and recovery process into 3 stages: (1) the sense-making and warning, (2) the coordinative response, and (3) the early recovery with cautiousness. The date when the first case was confirmed within the county (January 28) was used to divide the sense-making and coordination response stages, while the actions that occurred after all the infected cases were identified (February 10) are considered as early recovery efforts. All the critical information and essential local actions were reported in a timeline infographic, as shown in Table 2.

**Table 2.**
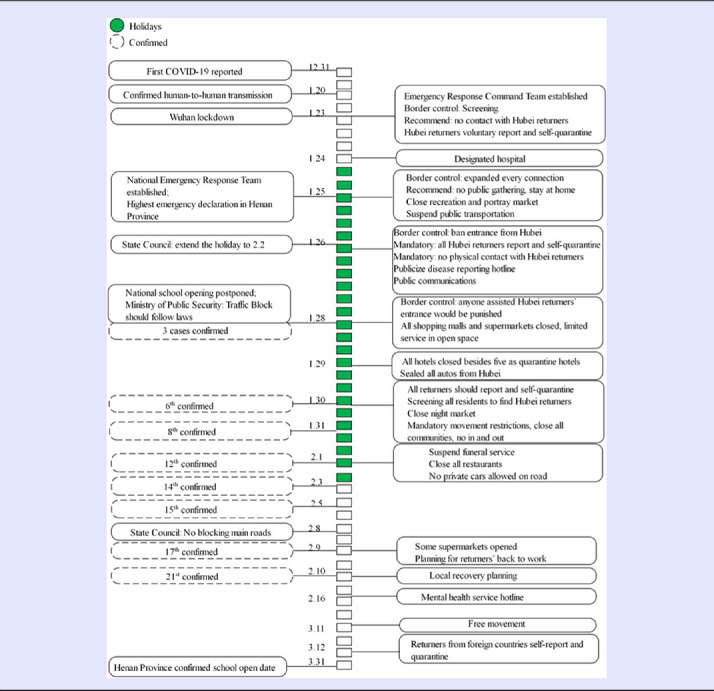
Timeline for important information and measures

## Sense-Making and Public Warning

Before Wuhan was put into lockdown (January 23), local news media and local TV reported the human-to-human transmission of the disease, as well as the first confirmed positive case in Henan province.

On January 23, the same day when Wuhan was put into lockdown, the T County government established a temporary emergency response and command team (referred to as ERCT in the following), and then officially established it on January 27, with the escalation of the outbreak. The ERCT structure is shown in Table 3. The Secretary of the Communist Party of China (CPC) of the county government was the director, and all the other leaderships of the county, including the mayor, vice mayors, vice CPC secretaries, were associate directors. Eight functional groups were established, and almost all the government agencies were involved. The mayor’s office led the Coordination group, the Health agency led the Medical Treatment group, the local CDC and Hospitals led the Technical Advisory group, the Public Communication agency led the Public Communication and Education group, the Market Regulation agency coordinated the Market Regulation and Price Monitoring group. The county’s Finance agency was in charge of the logistics and supply management, while the CPC’s self-discipline committee inspected all the government employees’ behaviors to make sure their activities were in line with laws and policies (Table 3).

**Table 3.**
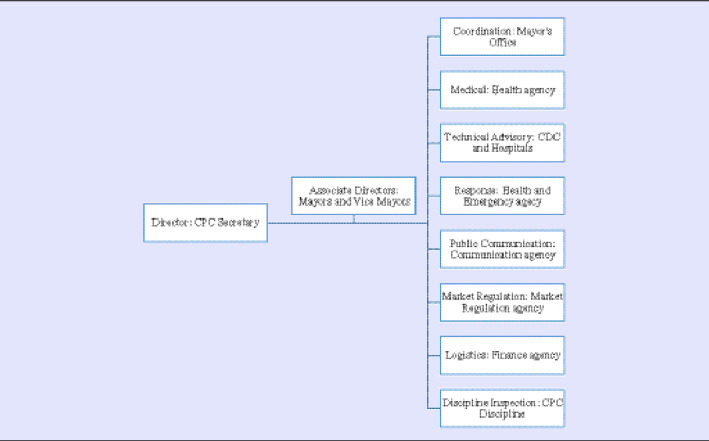
Command structure of the COVID-19 response team of T County

The local government’s ERCT immediately started to set border control check-points on January 23 when Wuhan declared it was locking down. It also issued an open letter to the public and potential returners from Wuhan and Hubei Province. The open letter recommended that (1) the potential returnees stay where they were; and (2) if they had returned, they should report to the local government voluntarily; and (3) if they felt sick, they should visit the public hospital that provides the free medical examination, and also recommended that (4) other local residents who had stayed in T County keep away from those who had recently returned. One day later, Henan Province declared the highest level of public health emergency declaration within the province, and the hospitals for COVID-19 treatment were designated across the province, at least 1 in each county.

On January 25, the day of the Spring Festival, the ERCT released 4 emergency orders for a potential outbreak. It should be underlined that the Chinese President, Xi Jinping, called for efforts of national response that same day, which was a critical factor influencing the local government’s actions. The 4 orders (1) suspended public transportations; (2) called all returnees from Hubei to report to the local authority; (3) called all residents to stop public gathering and celebration events; (4) closed all nonessential entertainment and leisure places, as well as live poultry markets; (5) set screening and check-points at every entrance of the county’ border; (6) provided a free medical examination for residents with potential symptoms; (7) set-up a special hotline for the public, and called the public to report any suspicious cases; and (8) called all government employees to get ready to get back to work.

**Figure 2.**
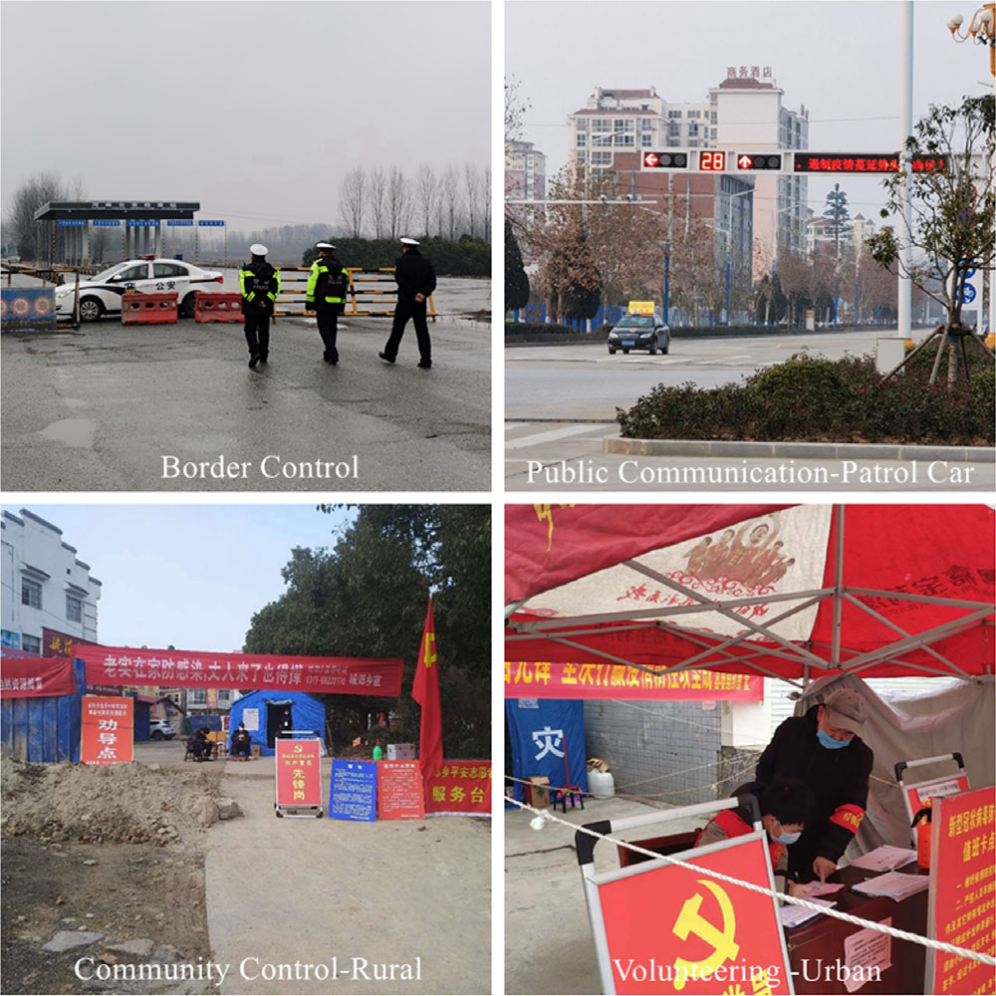
Social distancing measures and public communication (photos by the authors).

The social control measures escalated on January 26, while another 2 orders were released, and all the ERCT members were sent out county-wide to supervise the implementation of these orders. All new people returning from Hubei were refused to enter at check-points, and those who had already returned from Hubei were required to self-quarantined. Then, all communities and rural villages were recommended to be closed to restrict individuals’ nonessential movement, and every entrance and exit was to be recorded. Third, the school closure status was maintained until the overall situation would be judged to have become better. Fourth, public communication and warning strategies were established. All public communication channels were used to increase the public’s situation awareness. The government’s website, as well as social media accounts, became major outlets of official news and guidance. Local TV channel continued to broadcast the situation using subtitles, and mobile phone service carriers delivered 1 reminder message to all cell phones within the county every day. Meanwhile, the local broadcasting system in rural villages had to broadcast a situational update twice each day, and vehicles owned by government agencies were mounted with loudspeakers and sent out to the neighborhoods to blast the latest updates and protective recommendations (Figure 2, upper right corner).

## Coordinative Response

Three positive COVID-19 cases were confirmed on January 28, and the actions after were considered as the response efforts in this analysis. In the following 5 d, another 6 orders were released with stronger control measures. These measures included (1) closed all shopping malls, supermarkets, and restaurants, leaving only the sale of necessary living supplies (food and other essential goods) sold outside in open space; (2) sealed-up all autos from Hubei Province (cars with those plates were prevented from moving within the county), anyone caught assisting people from Hubei to come back were to be punished, and all returnees from other places were required to self-quarantined for 14 days; (3) all hotels were closed except for 5 hotels that were designated for quarantine; (4) all communities and villages were mandatorily closed with the most strict restrictions, and nobody was allowed outside for any nonnecessary or nonemergency issues, and every family could have 1 person go out for shopping in the designated places every 2 d; and (5) all funeral services were suspended.

Meanwhile, the local government started to use the Public Communication Briefs (PCB) as a policy tool, which notified the situation of COVID-19 spread within the county, the adjustments of the previously passed social restriction orders, the actions and efforts taken by local government, as well as the punishments for noncompliance behaviors.

In addition to the physical distancing measures, medical measures and health services were provided at the same time. The public hospital was designated as the special treatment for all fever-related symptoms. Medical examination and treatment for the COVID-19 infection were free for everyone. The local government assisted CDC experts in investigating the cases and in tracing the contacts. The logistic group managed to consolidate the supplies of personal protective equipment from multiple sources for the designated hospital, government employees, and community volunteers. On February 16, the county government even opened a hotline telephone number for mental health consultations with the help of doctors from local hospitals.

Moreover, several social service efforts were implemented to encourage the public’s compliance with physical distancing orders. These efforts included (1) encouraging online shopping and kept the delivery service/companies in function during the mobility restriction period; (2) providing shelters and necessary mass care services for the homeless; (3) recruiting volunteers for community controls; (4) sourcing medical supplies like masks from multiple sources, including both the formal suppliers, the higher level of government, the medical supply producers, and the help from the Business Association of T Residents who worked out of the county; (5) strengthened regulations on all government employees and made them come back to work much earlier than they had been scheduled to before the epidemic.

## Early Recovery with Cautiousness

It seems that the early recovery started from February 9, when the supermarkets re-opened, although all the infected cases were identified on February 10. The local business, including restaurants, hotels, and companies, re-opened gradually with limited service capacity, and take-out or online booking models were encouraged. The schools and recreation businesses remained closed. The internal movement restriction also loosened gradually, and the checking of temperature, keeping distance, and wearing masks became the primary measures.

Another significant effort made by the T County government was to conduct large-scale testing for the returnees who had to go back to work. The local government would provide a certificate for the returnees who accomplished 14 days of quarantine and had no positive testing results. Without these official certificates, the local government of planned destinations may refuse their entrance. To reduce the potential risks of new contaminations on the way back to cities using public transportations, the T government also worked closely with the destination cities that badly needed the reflux of workforce to organize collective traveling services from T to the big cities, and only the ones with negative testing results and 14 days of quarantine were allowed to travel.

All the traced contacts and suspected cases were tested, and positive COVID-19 cases were confirmed until February 10. On February 22, the last 10 patients who were positive but had not recovered yet were transferred to better hospitals in the higher (prefecture) level of governance. On March 1, all of the infected patients had recovered, and no death occurred in T County.

The freedom to move was re-allowed on March 11. Life seems to be back to normal since the middle of March, but there had been concern about 3 returnees recently back from Japan. The overall situation in China seemed to become better in late March but with uncertainties and the risk of a second outbreak because many Chinese citizens, as well as international travelers, are continuing to come back. “Pay attention to the returnees from foreign countries and the potential asymptomatic” is the current ongoing national strategy of response. This prudence also applies to T County’s local government.

After Wuhan’s re-opening on April 8, all China has gradually returned to the new normal life, living with the COVID-19 risk with alerts. Since then, several small local outbreaks occurred in Beijing, Xinjiang, Liaoning, Qingdao, and Shanghai recently, and all of these outbreaks were contained very quickly. Physical distancing, mass testing, contact tracing, and digital "health passport" are the primary measures for these after going outbreaks. Take the Qingdao outbreak in October as an example, the city government coordinated medical professionals and tested all the 10 million residents within 5 d.

## Limitations

We only analyzed 1 case study here to show how a local government responded to the COVID-19 outbreak. Comparison case studies should be conducted to generalize more reliable and applicable lessons for the ongoing COVID-19 pandemic.

## Discussion

In this study, we analyze a local government’s response to the COVID-19 outbreak in China using a framework including 3 stages, (1) sense-making and public warning, (2) coordinative response, and (3) early recovery with cautiousness, and 5 components: (1) emergency declaration and the establishment of the emergency response team, (2) the nonpharmaceutical interventions, (3) medical and health measures, (4) essential public service maintenance, and (5) public communications.

The nonpharmaceutical interventions and the physical distancing measures, in particular, were the primary methods used by the county government. Like previous modeling studies^6^ indicated, the implementation of strict physical distancing roles and the public’s compliance may be the most effective way of containing the spread of COVID-19, but with a substantial economic cost.^21^ Intergovernmental dynamics and a higher level of government’s actions can influence the behaviors of local governments.^22^ The county government’s behaviors were strongly influenced by the central government’s requirements and the local situation of the COVID-19 spread. After the confirmation of COVID-19 cases within the county, almost all the “voluntary” and “recommended” orders became mandatory, and the internal movement restriction escalated to the highest level with the increase of the confirmed cases. When all the suspected cases were tested and all contacts were traced, the restrictions started to loosen.

Chinese citizens are more compliant with regulations and have higher degrees of trust in their government and authorities than most of their peers in Western cultures.^10,23-25^ The timing of this outbreak is also vital for the public’s compliance. It occurred during the Spring Festival holiday, the longest holiday for families to stay together, and it was further extended. Most families had prepared food for several weeks during this holiday; thus, there was no worrying about necessary living supplies. Providing essential humanitarian and public services are parts of essential emergency functions,^26^ and they are also crucial for the public to comply and cooperate. The free medical examination, testing, and treatment for the COVID-19 infection played a critical role in encouraging the suspected cases and contacts to report to officials. Humanitarian assistance to the homeless, encouraging online shopping, and keeping necessary delivery service, as well as public sourcing for volunteers and donations, also facilitated the public’s compliance and eventually the emergency response to the COVID-19.

This study also finds the “Federalism” within the unitary government’s system during this pandemic: strict “border” control measures were widely implemented despite not having been called for by the central government. The border management on the provincial level was not prominent, but on the local level, most of the local government enacted strict border management to stop the movement of people. This was especially the case for preventing the entrance of people from Hubei, the most affected province. Pandemic emergency management and planning can raise extraordinary ethical dilemmas for local officials,^27^ especially for the entrance bans and potential stigmas^28^ caused when calling for the public to keep away from the suspected cases.

## Conclusions

This article analyzes a case of COVID-19 response at the level of a county government in China. The nonpharmaceutical interventions, especially the physical distancing measures, were the primary tools used to contain the spread of the virus. Medical and health countermeasures, maintenance of essential public services, and effective public communications tactics were important additions to the strict physical distancing measures, and they helped encourage the public’s compliance. The local government’s actions kept adapting to the situation of the local cases and the directions from the central government.
